# Carnosine alleviates diabetic nephropathy by targeting GNMT, a key enzyme mediating renal inflammation and fibrosis

**DOI:** 10.1042/CS20201207

**Published:** 2020-12-09

**Authors:** Xue-qi Liu, Ling Jiang, Lei Lei, Zhen-yong Nie, Wei Zhu, Sheng Wang, Han-xu Zeng, Shi-qi Zhang, Qiu Zhang, Benito Yard, Yong-gui Wu

**Affiliations:** 1 Department of Nephropathy, The First Affiliated Hospital of Anhui Medical University, Hefei, Anhui 230022, P.R. China; 2 Center for Scientific Research of Anhui Medical University, Hefei, Anhui 230022, P.R. China; 3 Department of Endocrinology, the First Affiliated Hospital of Anhui Medical University, Hefei, Anhui 230022, P.R. China; 4 Vth Department of Medicine (Nephrology/Endocrinology/Rheumatology) University Medical Center Mannheim, University of Heidelberg, Theodor-Kutzer-Ufer1-3, 68167 Mannheim, Germany

**Keywords:** Carnosine, Diabetic nephropathy, Fibrosis, Glycine N-methyltransferase, Inflammation

## Abstract

Diabetic nephropathy (DN) is a common microvascular complication of diabetes and the main cause of end-stage nephropathy (ESRD). Inflammation and fibrosis play key roles in the development and progression of diabetic nephropathy. By using *in vivo* and *in vitro* DN models, our laboratory has identified the protective role of carnosine (CAR) on renal tubules. Our results showed that carnosine restored the onset and clinical symptoms as well as renal tubular injury in DN. Furthermore, carnosine decreased kidney inflammation and fibrosis in DN mice. These results were consistent with high glucose (HG)-treated mice tubular epithelial cells (MTECs). Using web-prediction algorithms, cellular thermal shift assay (CETSA) and molecular docking, we identified glycine N-methyltransferase (GNMT) as a carnosine target. Importantly, we found that GNMT, a multiple functional protein that regulates the cellular pool of methyl groups by controlling the ratio of S-adenosylmethionine (SAM) to S-adenosylhomocysteine (SAH), was down-regulated significantly in the serum of Type 1 DM patients and renal tissues of DN mice. Moreover, using cultured TECs, we confirmed that the increased GNMT expression by transient transfection mimicked the protective role of carnosine in reducing inflammation and fibrosis. Conversely, the inhibition of GNMT expression abolished the protective effects of carnosine. In conclusion, carnosine might serve as a promising therapeutic agent for DN and GNMT might be a potential therapeutic target for DN.

## Introduction

Diabetic nephropathy (DN), a major microvascular complication of diabetes [1], is characterized by chronic microinflammation and excessive accumulation of extracellular matrix (ECM) proteins in glomeruli and the renal interstitium, both of which contribute to the progression of chronic renal failure [2–4]. Blockade of the renin–angiotensin-aldosterone system (RAAS) is currently the mainstay of treatment to delay the progress of DN [5]. Yet RAAS blockade is not curative and the reduction of proteinuria is insufficient in many patients to prevent further deterioration of renal function. This underscores the unmet demand for development of effective drugs that can be used in combination with RAAS blockade.

Carnosine is a dipeptide composed of β-alanine and L-histidine [6]. Studies have shown that carnosine has a strong antioxidant capacity, which can remove reactive oxygen radicals and α,β-unsaturated aldehydes formed by excessive oxidation of fatty acids in cell membranes during oxidative stress [7]. Recent studies suggested that administration of carnosine to experimental animals ameliorated acute renal failure caused by ischemia/reperfusion in rats [8]. In addition, administration of carnosine also suppressed renal sympathetic nerve activity in urethane-anesthetized rats [6,8]. However, whether carnosine exerts protective effects on DN, and the underlying molecular mechanism requires rigorous evaluation.

In the present study, we found that carnosine reduced STZ (streptozotocin)-induced renal tubular injury, kidney inflammation and fibrosis. Furthermore, we identified carnosine exert its renoprotective mechanism by targeting action on glycine N-methyltransferase (GNMT), a multifunctional and tissue-specific enzyme which controlling the ratio of S-adenosylmethionine (SAM) to S-adenosylhomocysteine (SAH) [9]. First, we found that GNMT was down-regulated significantly in the serum of Type 1 DM patients and renal tissues of DN mice. Moreover, using cultured TECs, we confirmed that GNMT was a newly identified enzyme mediating renal inflammation and fibrosis in DN. Therefore, carnosine might serve as a promising therapeutic agent and GNMT might be regarded as a potential target for treating DN.

## Materials and methods

### Chemicals and reagents

Carnosine and STZ were purchased from Sigma Chemical Company (St. Louis, MO, U.S.A.). The antibodies specific for anti-TNF-α and anti-β-actin were purchased from Santa Cruz Biotechnology (CA, U.S.A.). The antibodies Anti-KIM-1, Anti-P-P65, Anti-P65 and anti-cleaved caspase-3 were obtained from Cell Signaling (Danvers, MA, U.S.A.). Anti-GNMT, anti-COL-I, anti-α-SMA, anti-fibronectin and anti-E-cadherin were obtained from Abcam Biotechnology (Abcam, Cambridge, U.K.). Anti-SAM antibody (MA00201-50) was acquired from Acris Antibodies, Inc. (San Diego, CA, U.S.A.). Blood urea nitrogen (BUN), creatinine and Periodic acid–Schiff (PAS) Kits were acquired from Jiancheng Bioengineering Institute (Nanjing, Jiangsu, China). A Microalbumin Assay Kit was obtained from Abcam Biotechnology (Abcam, Cambridge, U.K.). One step TUNEL Apoptosis Assay Kit was acquired from Beyotime Institute (Nanjing, Jiangsu, China).

### STZ-induced diabetic mice model

All animal procedure protocols were conducted in SPF animal laboratory of Anhui Medical University. Animal maintenance were approved by the Ethics Committee of Animal Research of Anhui Medical University (Hefei, China) and conformed to the NIH Guide for the Care and Use of Laboratory Animals. Male C57BL/6J mice (approximately 6‒8 weeks) were purchased from the Experimental Animal Centre, Anhui Medical University. The experimental mice were kept in cages under standard conditions and were given free access to food and water in a room with a constant temperature of 22°C ± 2°C and a humidity of 60%. The light-dark period was 12 h. The mice were randomly separated into four groups (*n* = 6–8): Control, Control+ Carnosine, STZ, STZ+ Carnosine. After 7 days of adaptive domestication, STZ (Sigma, dissolved in 0.1 M citrate buffer, pH 4.5) was given daily to mice by intraperitoneal administration at a dose of 50 mg/kg after 4–6 h of food deprivation each day for five consecutive days. Carnosine was dissolved in drinking water volume at doses of 1 g of carnosine/kg per day for 8, 12 and 16 weeks. Water consumption and body weights of each cage were measured daily. Carnosine–supplemented drinking water were replaced every day. The 24-h urine samples were collected from the mice at the end of 16 weeks in metabolic cages. After inhaling 5% isoflurane anaesthesia, we collected mice blood samples in fasted state by heart punctures. Experimental animals were killed humanely after anaesthesia.

### Biochemical and physical analysis

The mice were fasted for 6 h and their blood glucose levels were measured with an Accu-Chek glucose meter (Roche diagnostics device) every 4 weeks as recommended by the Animal Models of Diabetic Complications Consortium. Body weight and kidney weight of each mouse were measured. Animal blood and urine samples were collected for biochemical analysis the day before kidney biopsy. Collected blood were centrifuged for testing of blood urea nitrogen (BUN) and creatinine. Urine albumin was measured using the ELISA Kit according to the manufacturer’s protocol.

### Kidney histology

Kidneys were taken out immediately and fixed in 4% paraformaldehyde 16 h. Fixed kidney samples were first embedded with paraffin and then cut into 4-μm-paraffin sections. After deparaffinization, to identify kidney structures, we used PAS staining of paraffin sections for histology analysis, and examined by microscope (Zeiss AX10 microscope, Carl Zeiss Canada Ltd, Canada) at ×400 magnification. Ten fields were randomly selected to evaluate and grade mesangial expansion index and tubulointerstitial injury index. For immunohistochemistry, the samples were treated with 3% hydrogen peroxide and microwave heated at 95°C for 20 min to block the activity of endogenous peroxidase and antigen. The tissue sections were sealed with goat serum at 37°C for 30 min and then incubated with anti-TNF-α, anti-α-SMA, anti-GNMT antibodies for 24 h at 4°C and secondary antibodies for 30 min at 37°C. According to the manufacturer’s instructions, the tissue sections were stained by Masson’s Trichrome staining reagent (Zhuhai Besso Biotechnology Institute, China). The slides were visualized after staining with DAPI for 5 min under a microscope.

### Cell culture

Mice tubular epithelial cells (MTEC) were provided by Professor Huiyao Lan from the Chinese University of Hong Kong. The experimental cells were all between passages 6‒15. Cells were cultured in low-glucose DMEM (Gibco, CA) with 10% FBS (Gibco, CA) at 37°C in an atmosphere containing 5% CO_2_. The cells were treated with high glucose DMEM (30 mM glucose) and mannitol (5.5 mM glucose + 24.5 mM mannitol) containing 0.1% FBS. Meanwhile, cells were incubated with carnosine or the same amount of PBS for 24 h for further study.

### MTT assay

MTEC cells were subcultured in 96-well plates and treated with different concentrations of carnosine for 24 h and then added in high glucose DMEM (30 mM glucose) for 24 h. At last, we added 5 mg/ml of MTT solution and incubation for 4 h. The absorbance of 96-well plates was determined by the microplate reader (Multiskan MK3, Thermo, U.S.A.) at the wavelength of 550 nm for measurements of optical density (OD).

### Transmission electron microscopy

MTEC cells were added with 0.1 M cacodylate sodium (pH 7.4) to 2.5% glutaraldehyde and fixed at 4°C for 72 h. The cells were incubated at room temperature with 2% osmium tetroxide and 0.1 M cacodylate sodium (pH 7.4) for 1 h. Polymerization was achieved in gelatin capsules at 60°C for 48 h. The specimens were then examined with transmission electron microscope (H-7700, Tokyo, Japan).

### GNMT knockdown and overexpression in MTEC cells by transfecting shRNA

MTEC cells were transfected with GNMT shRNA (Genechem, Shanghai, China) by mixing Lipofectamine TM 3000 reagent (Invitrogen) according to the manufacturer’s instruction. The negative scrambled shRNA was used as a control. We combined and incubated the diluted shRNA and Lipofectamine 3000 for 20 min at 37°C in the dark atmosphere, the mixture was then added to the cells. After 6–8 h of incubation, the cells were grown in low glucose DMEM containing 10% FBS. Lentivirus GNMT and vector were constructed by Genechem Biotechnology (Shanghai, China). Lentivirus GNMT or vector (multiplicity of infection, MOI = 10) was transfected into MTEC cells. About 5 μg/ml polybrene (Genechem, Shanghai, China) was added in culture medium to improve transfection efficiency. After transfection for 48 h, 2 μg/ml puromycin (Solarbio, China) was mixed with culture medium. The stable transfected cells were screened for 1 week. The survival cells were acted as stable GNMT-overexpressing MTEC cells. Real-time PCR and Western blot were used to measure the overexpression and knockdown efficiency of GNMT.

### RNA isolation and real-time PCR

RNA was extracted with TRIZOL lysis buffer (Invitrogen, CA), and NanoDrop2000 spectrophotometer (Thermo Fisher Scientific, MA) was used to determine the concentration and purity of RNA. After unifying the sample mass, the sample volume was calculated according to the sample concentration. The RNA was denatured to cDNA using a reverse transcriptome. The primers were designed for mRNAs. Real-time PCR assay was run by CFX96 real-time PCR system (Bio-Rad, CA) with SYBR Premix Ex Taq™ II (Takara, Japan). The primers used were shown in Table 1. PCR amplification was performed for more than 40 cycles under the conditions of denaturation at 95°C for 20 s, annealing at 58°C for 20 s, and elongation at 72°C for 20 s. The mRNA expression value was normalized to the β-actin expression value using the 2^−ΔΔCT^ method.

**Table 1:** Sequences of primers

Genes	Forward (5′–3′)	Reverse (5′–3′)
Mouse IL-1β	CTTTGAAGTTGACGGACCC	TGAGTGATACTGCCTGCCTG
Mouse GNMT	GTTGACGCTGGACAAAGA	AGCCTGTGCTGAGGATA
Mouse TNF-a	CATCTTCTCAAAATTCGAGTGACAA	TGGGAGTAGACAAGGTACAACCC
Mouse β-actin	GCGTGACATCAAAGAGAAGC	GCGTGACATCAAAGAGAAGC

### Western blot

We used RIPA buffer (Beyotime, Jiangsu, China) to extract renal tissue proteins or cellular proteins, and then quantified their concentration using the BCA Kit (Beyotime, Jiangsu, China). Proteins (30 μg) were segregated via 10% sodium dodecyl sulfate polyacrylamide gel electrophoresis and were transfer to nitrocelluose membrane. Seal with 5% skimmed milk for 1–2 h, wash and incubate with the corresponding primary antibody for 24 h. The secondary antibody (Rockland Immunochemicals, U.S.A.) was incubated again for half an hour. The intensities of the band were detected by the enhanced chemiluminescence detection system (Bio-Rad, CA). Finally, ImageJ (NIH, Bethesda, U.S.A.) was used to quantitatively analyze the protein bands and normalization to β-actin.

### Carnosine concentration

Carnosine concentrations were measured by A UPLC Dionex Ultimate 3000 chromatographic system (Thermo Scientific, San Jose, U.S.A.) consisting of binary pump, degasser, autosampler and column oven coupled to a Q-Exactive plus hybrid quadrupole-orbitrap mass spectrometer (Thermo Scientific, San Jose, U.S.A.) with a heat electrospray ionization (HESI) through Targeted selected ion monitoring (T-SIM) mode. The carnosine stock solution was dissolved in water, the calibration line was prepared at seven spiking levels (1, 5, 10, 20, 50, 100 and 200 ng/ml) by diluting stock solution with water. An aliquot of 20 μl serum (*n*=6–8) or 100 µl renal sample homogenate (*n*=6–8) was mixed with 50 or 100 µl cold acetonitrile (Thermo Scientific, San Jose, U.S.A.) for protein precipitation. After vortex 30 s, the sample was proceeded with high speed centrifugation (12000×***g*** for 20 min at 4°C). The supernatant was finally filtered with a 0.22 µm syringe filter before use. The separation was performed in a Waters Acquity UPLC BEH Amide column (100 mm × 2.1 mm, 1.7 µm, Waters, U.S.A.). Isocratic mobile solvents were consisted of acetonitrile : water (containing 0.1% formic acid) = 7:3, The flow rate was 0.3 ml/min, and the injection volume was 10 µl, the column temperature maintained at 45°C. Xcalibur 4.1 software (Thermo Fisher Scientific, San Jose, U.S.A.) was used to control the instrument and for data acquisition and analysis.

### Immunofluorescence assay

The MTEC cells were grown on slides first and then fixed with 4% acetone at room temperature for 10 min. We blocked cells with 10% BSA (bovine serum albumin) at 37°C. The primary antibody was incubated overnight. After washing with PBS three times, the goat anti-rabbit IgG-rhodamine (Bioss, Beijing, China) antibody was added and incubated for 1 h in the dark room at room temperature. DAPI was incubated for 5 min to stain the nuclei. After washed the cells for three times, the slides were imaged with the inverted fluorescence microscope (Zeiss Spot; Carl Zeiss Canada Ltd, Canada).

### Flow cytometry

Cells were washed with PBS three times, and then resuspended in binding buffer. After incubated with 10 µl of Annexin V-FITC and 5 µl of PI, cells were analyzed on BD FACSVerse flow cytometry (BD FACSVerse; BD Biosciences, Franklin Lakes, NJ, U.S.A.). Using Annexin V+/PI− (early apoptosis) and Annexin V+/PI+ (late apoptosis) to express the rate of apoptosis.

### Molecular docking

Molecular docking was adopted to understand the potential interactions between carnosine and GNMT. Discovery Studio 2017 R2 (BIOVIA Software, Inc., San Diego, CA, U.S.A.) software was employed in the present study. The structure of carnosine was optimized by Minimize protocol. The X-ray crystal structure of GNMT (PDB ID: 3THR) was downloaded from the RCSB Protein Data Bank. GNMT were prepared by Prepare protein protocol. CDOCKER protocol was applied to run molecular docking. Other parameters were set as default.

### Cellular thermal shift assay

Treating MTEC cells with or without carnosine for 24 h, and then we extracted cellular proteins. The samples were adjusted to similar concentrations using the BCA Kit. The same samples were placed in different PCR tubes and denatured for 8 min at different temperatures on a PCR machine (Eppendorf, Germany). After the protein was centrifuged, it was frozen in liquid nitrogen, and the supernatant protein was detected by Western blot.

### TUNEL assay

MTEC cell apoptosis was examined by TUNEL staining using the One step TUNEL Apoptosis Assay Kit. The cells were exposed to a TUNEL reaction mixture containing TM red–labeled dUTP. The TUNEL positive nuclei were examined by fluorescence microscopy (Zeiss AX10 microscope, Carl Zeiss Canada Ltd, Canada).

### GNMT activity measurement

GNMT activity of MTEC cells and kidney were measured by activated carbon adsorption according to the manufacturer’s protocol (R&D systems, 6526-MT-010).

### Patients

The protocol of clinical research was approved by the ethics committee of The First Affiliated Hospital of Anhui Medical University (Hefei, China) and conformed to the international standards (US Federal Policy for the Protection of Human Subjects). We selected Type 1 DM patients from Department of Nephropathy, the First Affiliated Hospital of Anhui Medical University. Inclusion criteria are as follows: (1) diagnosed as Type 1 diabetes; (2) 24-h urine albumin protein >300 mg/24 h; (3) diagnosis of DN by renal biopsy; (4) no obvious signs of apyrexial and infection, no cancer, no autoimmune disease. After obtaining the consent of patient and ethics committee, serum from healthy volunteers (*n*=6) and Type 1 DM patients (*n*=6) were collected in the morning after fasting for 12 h and processed within 6 h after collection. Characteristics of healthy volunteers and Type 1 DM patients were described in Table 2.

**Table 2:** Gender and age of all volunteers

Rank	Age	Gender
Control 1	25	Female
Control 2	26	Female
Control 3	24	Female
Control 4	25	Male
Control 5	24	Male
Control 6	26	Male
Type1 DN Patient 1	35	Female
Type1 DN Patient 2	42	Female
Type1 DN Patient 3	39	Male
Type1 DN Patient 4	48	Male
Type1 DN Patient 5	42	Male
Type1 DN Patient 6	53	Male

### Enzyme-linked immunosorbent assay (ELISA)

The protein levels of GNMT, TNF-α and IL-1β were tested by ELISA Kit (Jianglai Biotechnology Co., LTD, Shanghai, China) according to the product protocols.

### Statistical analyses

All experiments were conducted independently for three times. *P*<0.05 was regarded as statistically significant difference and was expressed as mean ± SD. Statistical significance of differences was determined by Student’s two-tailed *t*-test or one-way analysis of variance using GraphPad Prism 5 software.

## Results

### Carnosine restores HG-induced cell injury in tubular epithelial cells

**Figure 1:**
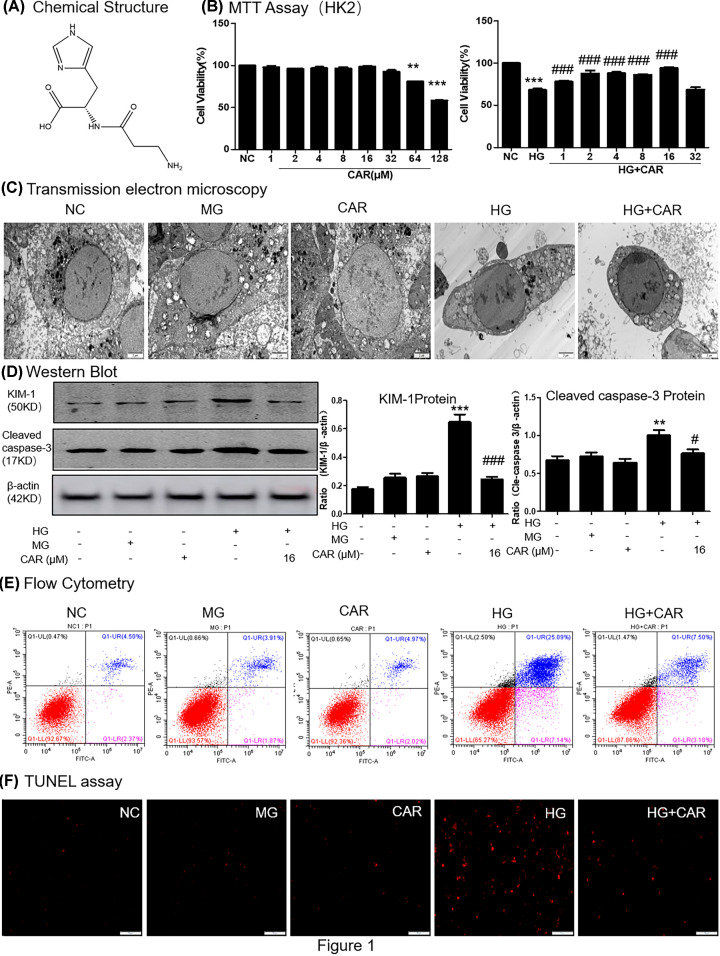
Effect of carnosine on HG-treated MTECs viability (**A**) The molecular structural formula of carnosine. (**B**) MTT assay of carnosine on MTECs viability and HG-treated MTECs cell viability. (**C**) Representative transmission electron microscopy; scale bar = 2 μm. (**D**) Western blot of KIM-1 and cleaved caspase-3 in MTECs. (**E**) Flow cytometry of PI/Annexin-V in MTECs. (**F**) TUNEL assay in MTECs. Results represent means ± SEM for three independent experiments.**P<0.01, ***P<0.001 vs NC. #*P*<0.05, ###*P*<0.001 vs HG; Abbreviation: MG, 5.5 mM glucose plus mannitol 24.5 mM mannitol; HG, high glucose; CAR, carnosine.

The molecular structure of carnosine was shown in Figure 1A. Use the MTT method to determine the cytotoxicity of carnosine and screen the optimal concentration for the next experiment (Figure 1B). The concentration of carnosine was less than 64 μM demonstrated a minimal effect on MTEC cell viability. In addition, when carnosine at concentration of 16 μM had the best effect to restore the viability of HG-treated cells. Results of transmission electron microscopy (TEM) analyses showed that carnosine protected MTEC cells from HG-induced cell injury and apoptosis (Figure 1C). Furthermore, we assessed the effect of carnosine by detecting the changes of KIM-1 and cleaved caspase-3 protein levels, results showed that they were markedly down-regulated by treatment of carnosine (16 μM; Figure 1D). We used flow cytometry of PI/Annexin V staining to detect MTEC cell death (Figure 1E). The flow cytometry data suggested that carnosine alleviated HG-induced cell apoptosis. Moreover, TUNEL staining showed that carnosine reduced apoptotic levels (Figure 1F).

### Carnosine alleviates the HG-induced inflammation response and ECM accumulation

**Figure 2:**
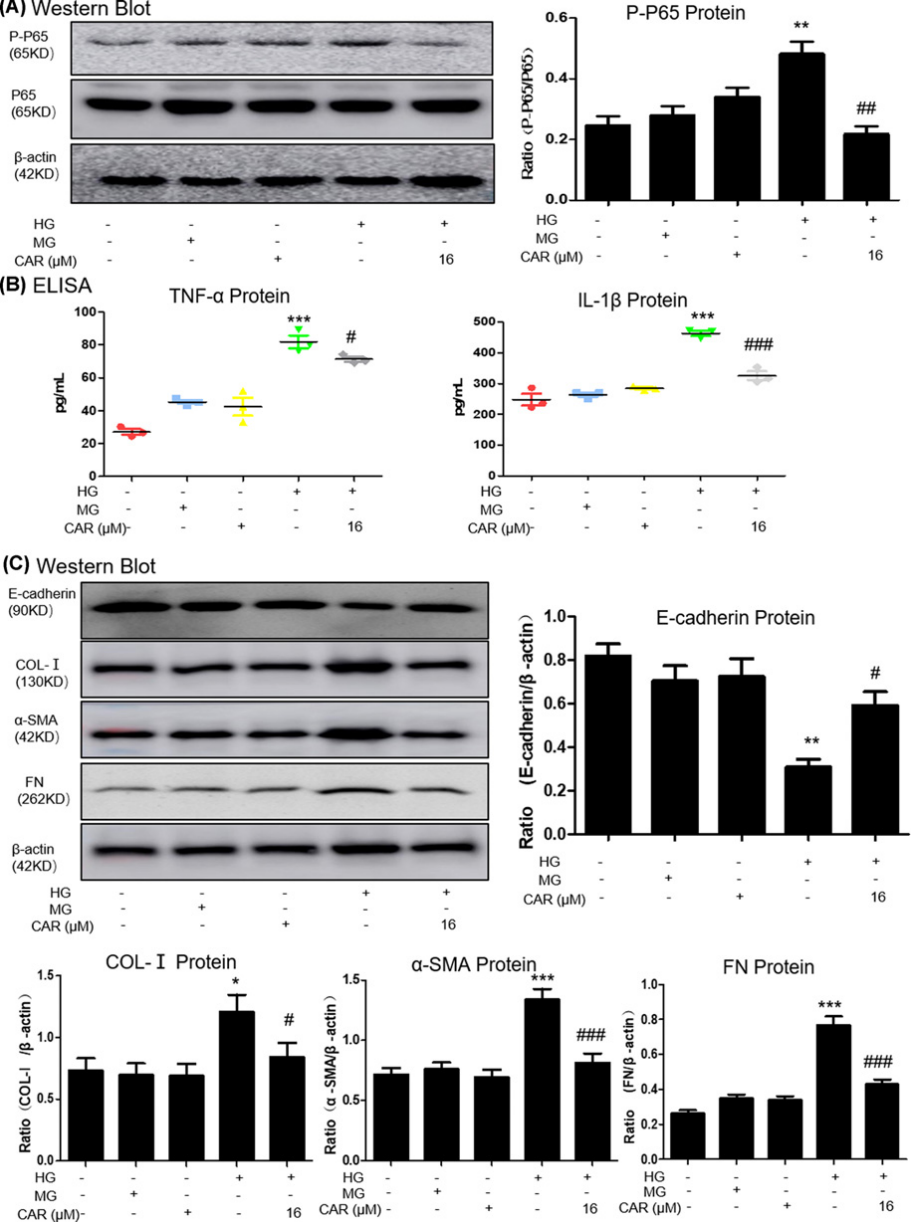
Carnosine inhibits HG-induced inflammation and pro-fibrotic responses (**A**) Western blot analysis of P-P65 in MTECs. (**B**) ELISA of TNF-α and IL-1β in MTECs. Results represent means ± SEM for three independent experiments. (**C**) Western blot of Col-I, α-SMA, FN and E-cadherin in MTECs. Results represent means ± SEM for three independent experiments. *P<0.05, **P<0.01, ***P<0.001 vs NC. #*P<*0.05, ##*P<*0.01, ###*P<*0.001 vs HG. Abbreviation: MG, 5.5 mM glucose plus mannitol 24.5 mM mannitol. HG, high glucose. CAR, carnosine.

Results of Western blot detected that carnosine treatment reduced the level of p65 NF-κB phosphorylation (Figure 2A). Moreover, ELISA analysis showed that carnosine reduced the levels of inflammatory markers such as TNF-α and IL-1β. (Figure 2B). To further confirm the ECM accumulation of MTEC cells, the expressions of E-cadherin, collagen I, a-SMA and fibronectin were detected. Results from Western blot suggested that carnosine treatment suppressed HG-induced ECM accumulation (Figure 2C).

### Carnosine target prediction

**Figure 3:**
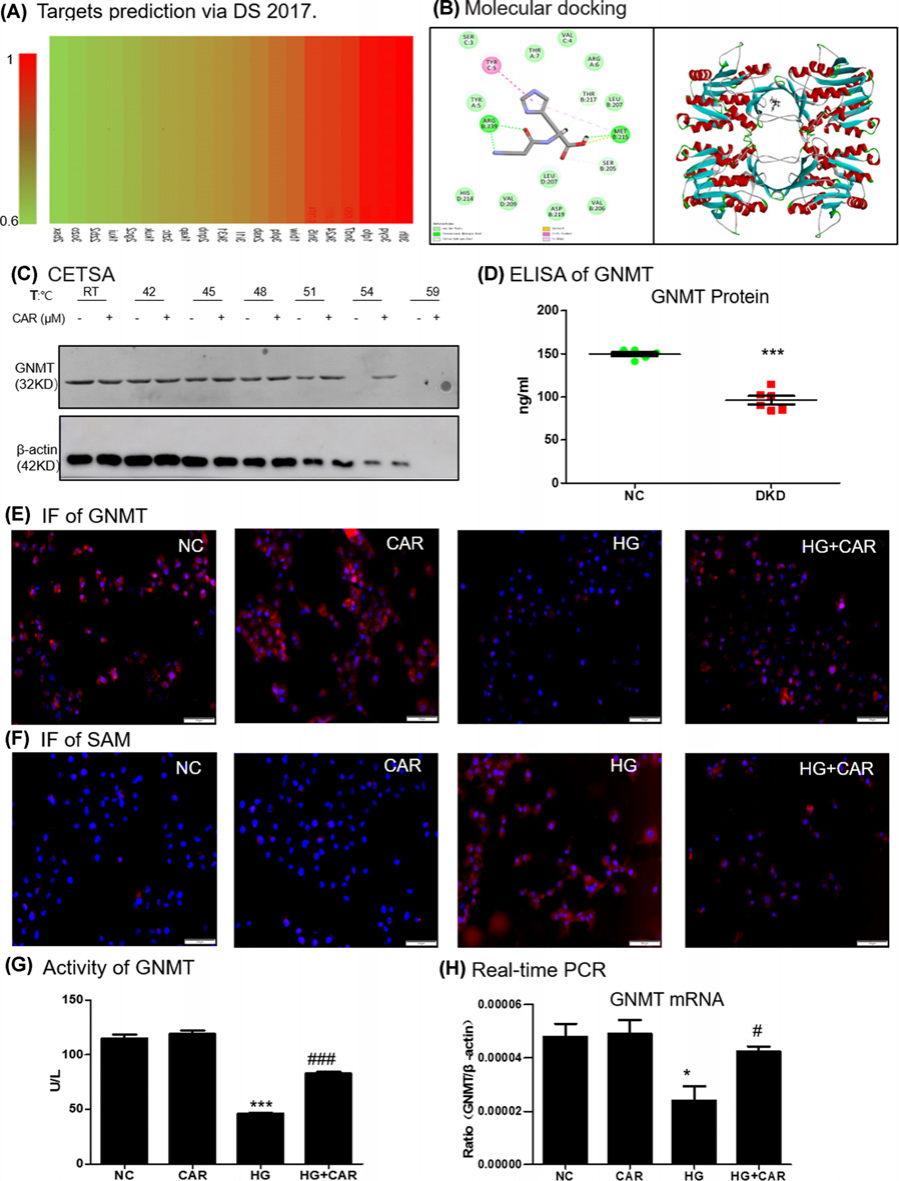
Prediction of carnosine molecular targets (**A**) Profiling of the predicted protein targets of carnosine via DS 2017. (**B**) Molecular docking of carnosine binding to GNMT crystal structure. (**C**) CETSA analysis in MTECs. (**D**) ELISA of GNMT in serum of healthy volunteers (*n*=6) and Type 1 DN patients (*n*=6). (**E**) Immunofluorescence of GNMT (red). Nuclei were counterstained with DAPI (blue). (**F**) Immunofluorescence of SAM (red). Nuclei were counterstained with DAPI (blue). (**G**) Activity of GNMT in MTECs. (**H**) Real-time PCR of GNMT in MTECs. Results represent means ± SEM for three independent experiments. *P<0.05, ***P<0.001 vs NC. #*P<*0.05, ###*P<*0.001 vs HG; scale bar = 100 μm. Abbreviations: CAR, carnosine;; HG, high glucose; NC, normal control.

Target prediction of carnosine using Discovery Studio 2017 (DS 2017) software. As shown in Figure 3A, the binding strength of carnosine and potential targets decreased from red to green. The fitting value indicated the score of the predicted targets, and the ten targets with the highest fitting value were shown in Table 3. Among these predicted binding target proteins, GNMT had a high fitting value of 0.9354.

**Table 3:** Top ten putative protein targets of carnosine

Rank	PDB ID	Putative target	Fit value
**1**	3tlh	Glycine N-methyltransferase	0.9354
**2**	3cyq	Chemotaxis protein motB	0.9269
**3**	1qfo	PROTEIN (SIALOADHESIN)	0.9205
**4**	3nd7	Phosphopantetheine adenylyltransferase	0.9157
**5**	3k24	Cathepsin L1	0.9028
**6**	3hl5	Baculoviral IAP repeat-containing protein 4	0.8946
**7**	1biw	PROTEIN (STROMELYSIN-1 COMPLEX)	0.8832
**8**	3ptq	Beta-glucosidase Os4BGlu12	0.8617
**9**	2xsb	HYALURONOGLUCOSAMINIDASE	0.8530
**10**	3i1l	Hemagglutinin-esterase protein	0.8214

### Carnosine binds directly to GNMT in HG-treated MTEC cells

Molecular docking was performed to study the binding mode between carnosine with GNMT (PDB ID: 3THR). The binding mode was displayed by 2D and 3D diagrams. The highest docking score provided by -CDOCKER_INTERACTION_ENERGY was 36.5045 kcal/mol. As shown in Figure 3B, three conventional H bond with MET215 and ARG239, two C-H bond with SER205 and THR217 were generated between carnosine and GNMT. Furthermore, other binding interactions, such as pi-pi stacked with TYR5, sulfur-X with MET215, and pi-alkyl with MET215, contributed to the binding affinity.

In order to assess target engagement, we also verified the interaction between carnosine and GNMT protein by performing a CETSA. The results showed that after treatment with or without carnosine, the denaturation temperature of GNMT was different in the range of 50–60°C. The GNMT cells after carnosine treatment had significantly higher thermal stability, indicating that carnosine directly binds to GNMT protein (Figure 3C).

Interestingly, we tested the release of GNMT by collecting serum from healthy volunteers (*n*=6) and type 1 DN patients (*n*=6). The results showed that the serum GNMT content of DN patients was also significantly reduced (Figure 3D).

### Carnosine enhances GNMT signaling in HG-treated MTEC cells

Bioinformatics Prediction sites suggested that carnosine may target GNMT. Immunofluorescence results showed that GNMT level was significantly up-regulated in response to carnosine therapy (Figure 3E). Conversely, SAM levels, the substrate for GNMT enzyme, decreased significantly (Figure 3F). We further confirmed that carnosine treatment increased the GNMT activity in HG-treated MTEC cells (Figure 3G). Additionally, the decreased mRNA level of GNMT in HG-treated MTEC cells, however, was markedly reversed by carnosine treatment (Figure 3H).

### Carnosine attenuates HG-Induced cell death, inflammation and ECM accumulation through GNMT-dependent mechanisms

**Figure 4:**
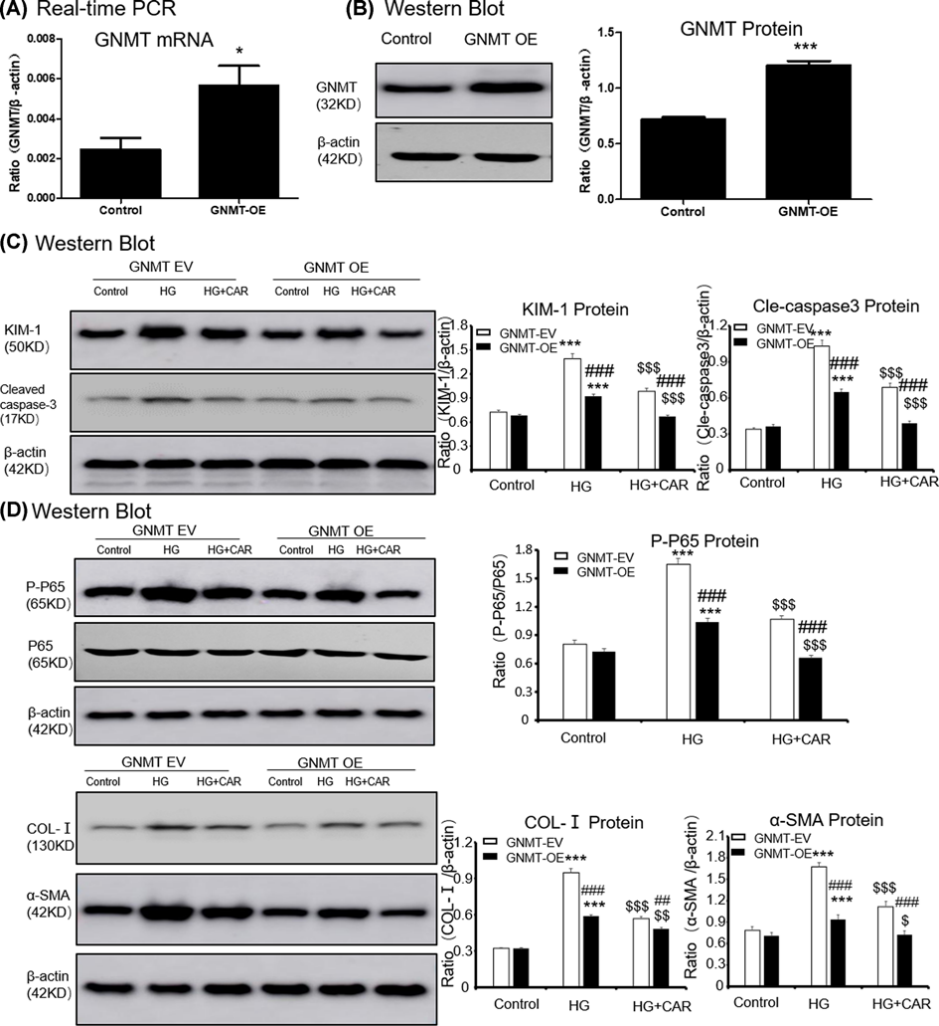
Overexpressed GNMT and treatment with carnosine have a similar cellular protective role (**A**) Real-time PCR of GNMT in MTECs. (**B**) Western blot of GNMT in MTECs. (**C**) Western blot of KIM-1 and cleaved caspase-3 in MTECs. (**D**) Western blot of p65 NF-κB phosphorylation, Col-I and α-SMA in MTECs. Results represent means ± SEM for three independent experiments. *P<0.05, ***P<0.001 vs NC. #*P*<0.05, ###*P*<0.001 vs GNMT-OE group. *$P*<0.05, *$$P*<0.01, *$$$P*<0.001 vs HG. Abbreviations: HG, high glucose. CAR, carnosine. EV, empty vector; OE, overexpression.

First, we overexpressed GNMT using lentivirus (Figure 4A,B), and the results showed that the overexpressed GNMT and treatment with carnosine play a similar cellular protective role in suppressing KIM-1, cleaved caspase-3 levels, inflammation and ECM accumulation (Figure 4C,D). Moreover, after overexpressing GNMT, treatment with carnosine had a superposition effect to reduce cell injury.

**Figure 5:**
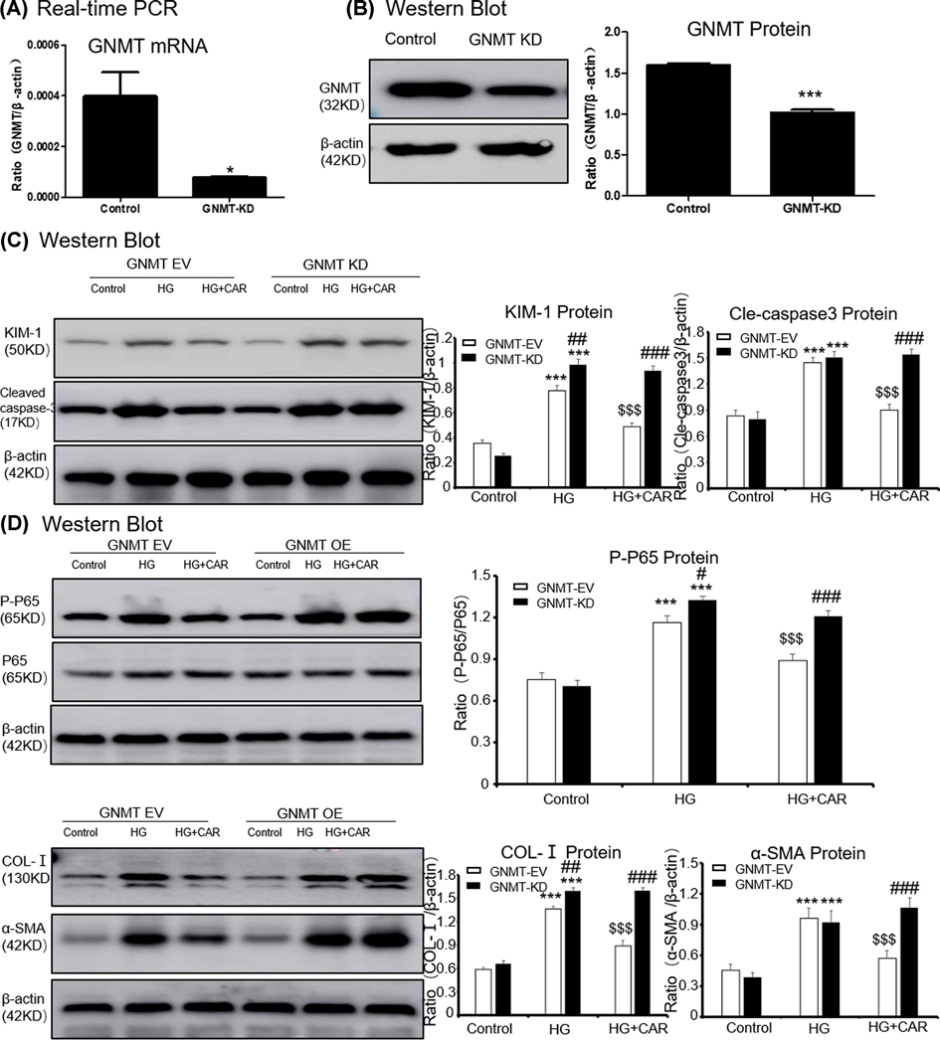
Carnosine fails to reduce the HG-induced cell death, inflammatory response and cell ECM in GNMT-silenced MTEC cells (**A**) Real-time PCR of GNMT in MTECs. (**B**) Western blot of GNMT in MTECs. (**C**) Western blot of KIM-1 and cleaved caspase-3 in MTECs. (**D**) Western blot of p65 NF-κB phosphorylation, Col-I and α-SMA in MTECs. Results represent means ± SEM for three independent experiments. *P<0.05, ***P<0.001 vs NC. #*P*<0.05, ##*P*<0.01, ###*P*<0.001 vs GNMT-KD group. *$$$P*<0.001 vs HG. Abbreviations: HG, high glucose; CAR, carnosine; EV, empty vector; KD, knockdown.

Furthermore, the GNMT in MTEC cells was knocked down by GNMT shRNA (Figure 5A,B). When GNMT was inhibited, carnosine could not further suppress KIM-1 and cleaved caspase-3 levels (Figure 5C). Results showed that inhibition of GNMT expression could increase HG-induced P65 phosphorylation and NF-κB mediated inflammatory response significantly. However, in the case of GNMT knockdown, carnosine was no longer able to continue to play its cellular protective role (Figure 5D). Importantly, we found that carnosine could not inhibit the accumulation of ECM after GNMT knockdown, suggested that carnosine mainly play a role by targeting GNMT (Figure 5D).

### Carnosine alleviates clinical symptoms and kidney injury in STZ-induced experimental mice model of Type 1 diabetes

**Figure 6:**
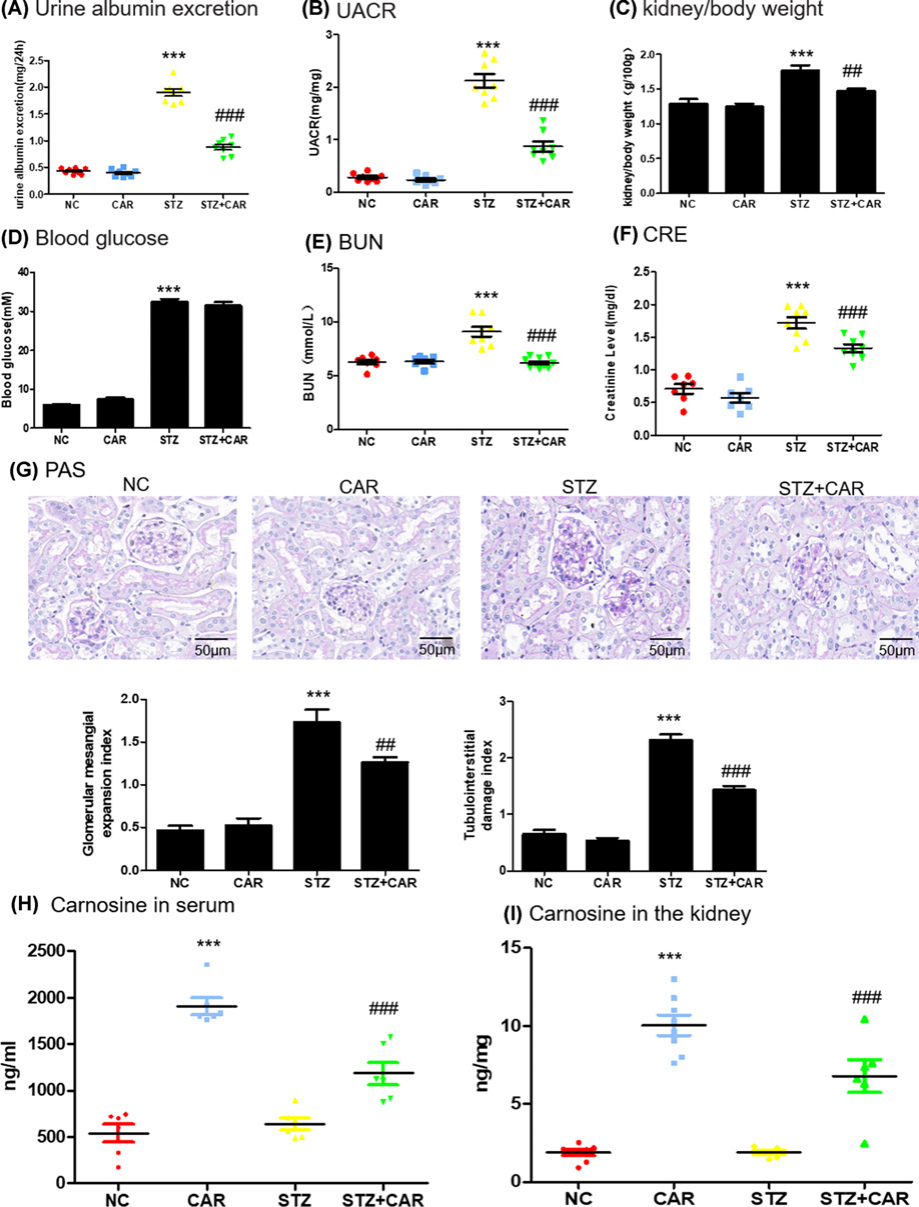
Physical and biochemical markers and pathology in DN mice (**A**) Analysis of urine albumin excretion. (**B**) Analysis of urinary albumin-to-creatinine ratio (UACR). (**C**) Kidney/body weight. (**D**) Blood glucose. (**E**) serum BUN assay. (**F**) serum Cr assay. (**G**) Histological observations of kidney sections stained with PAS. (**H**) The concentration of carnosine in serum. (**I**) The concentration of carnosine in mice kidney. Results represent means ± SEM for 6–8 mice. ***P<0.001 vs NC. ###*P<*0.001 vs STZl; scale bar = 100 μm. Abbreviations: NC, normal control; CAR, carnosine; BUN, blood urea nitrogen; Cr, creatinine.

In order to test whether carnosine can protect kidney in DN mice, diabetic mice and control mice were fed with carnosine (1 g/kg body weight). Diabetic mice showed polydipsia, polyuria and polyphagia accompanied by significant weight loss, hair color messy and dirty, and slow movement. But these symptoms improved significantly in mice treated with carnosine. As shown in Figure 6A and Supplementary Figure S1A, in 8-, 12- and 16-week diabetic mice, albuminuria was significantly increased in an age-dependent manner and decreased after carnosine treatment. We found that the increased urinary albumin-to-creatinine ratio (UACR) in the diabetic mice was markedly reduced by carnosine treatment (Figure 6B). The results also showed that the kidney weight ratio was significantly reduced after carnosine treatment (Figure 6C). The blood glucose level of STZ injection group was notably higher than that of control group. However, carnosine treatment could not reduce the blood glucose levels (Figure 6D and Supplementary Figure S1B). Carnosine improved renal function, as evidenced by alleviating serum creatinine and BUN values (Figure 6E,F). Histological analysis of kidneys stained with periodic acid–Schiff (PAS) showed that carnosine treatment reduced the glomerular mesangial expansion index and tubulointerstitial damage index in diabetic mice (Figure 6G). Furthermore, the concentrations of carnosine in serum or kidney were shown in Figure 6H,I and Supplementary Figure S1C and D. Results showed that after carnosine treatment, renal and serum carnosine levels increased significantly, but not in an age-dependent manner.

### Carnosine ameliorates STZ-induced down-regulation of GNMT and inhibits apoptosis

**Figure 7:**
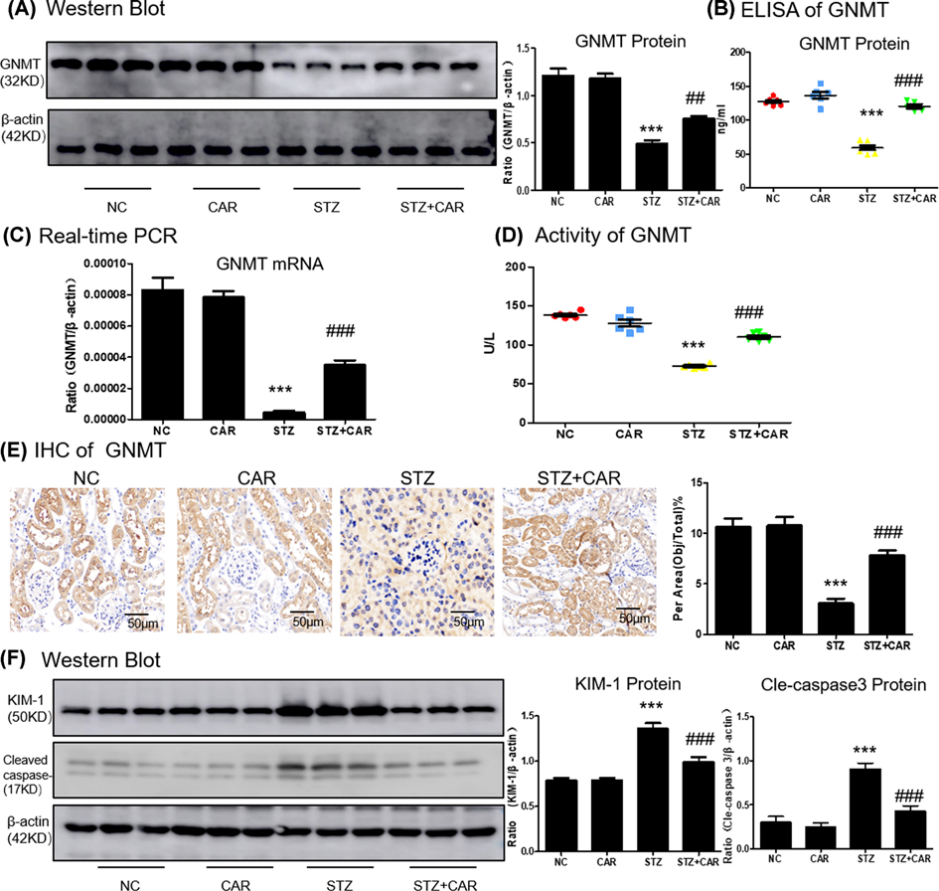
Carnosine decreases GNMT level and cell death in DN mice (**A**–**C**) Western blot, ELISA and real-time PCR of GNMT in mice kidney. (**D**) Activity of GNMT in mice kidney. (**E**) Immunohistochemistry of GNMT in mice kidney. (**F**) Western blot of KIM-1 and cleaved caspase-3 in mice kidney. Data represent the mean ± SEM for 6–8 mice. Results represent means ± SEM for 6–8 mice. ***P<0.001 vs NC. ##*P<*0.01, ###*P<*0.001 vs STZ; scale bar = 100 μm. Abbreviations: NC, Normal control; CAR, carnosine.

We predicted whether carnosine affects GNMT in DN mice by Western blot and ELISA. Results suggested that the GNMT levels decreased in response to DN mice but increased on treatment with carnosine (Figure 7A,B). Additionally, the decreased mRNA level of GNMT in Type 1 diabetes mice was markedly reversed by carnosine treatment (Figure 7C). We further confirmed that carnosine treatment increased the GNMT activity in diabetes mice (Figure 7D). To determine the expression quantity and location of GNMT, the results of IHC indicated that GNMT was mainly expressed on renal tubules, and carnosine increased GNMT levels in DN mice (Figure 7E). Results also showed significantly upregulation of KIM-1 and apoptosis-correlated cleavage of caspase-3 induced by STZ that decreased upon carnosine treatment (Figure 7F). Thus, these results suggested the protective effects of carnosine on DN-caused kidney injury.

### Carnosine protects against STZ-induced experimental mice model of Type 1 diabetes by ameliorates inflammatory response and fibrosis

**Figure 8:**
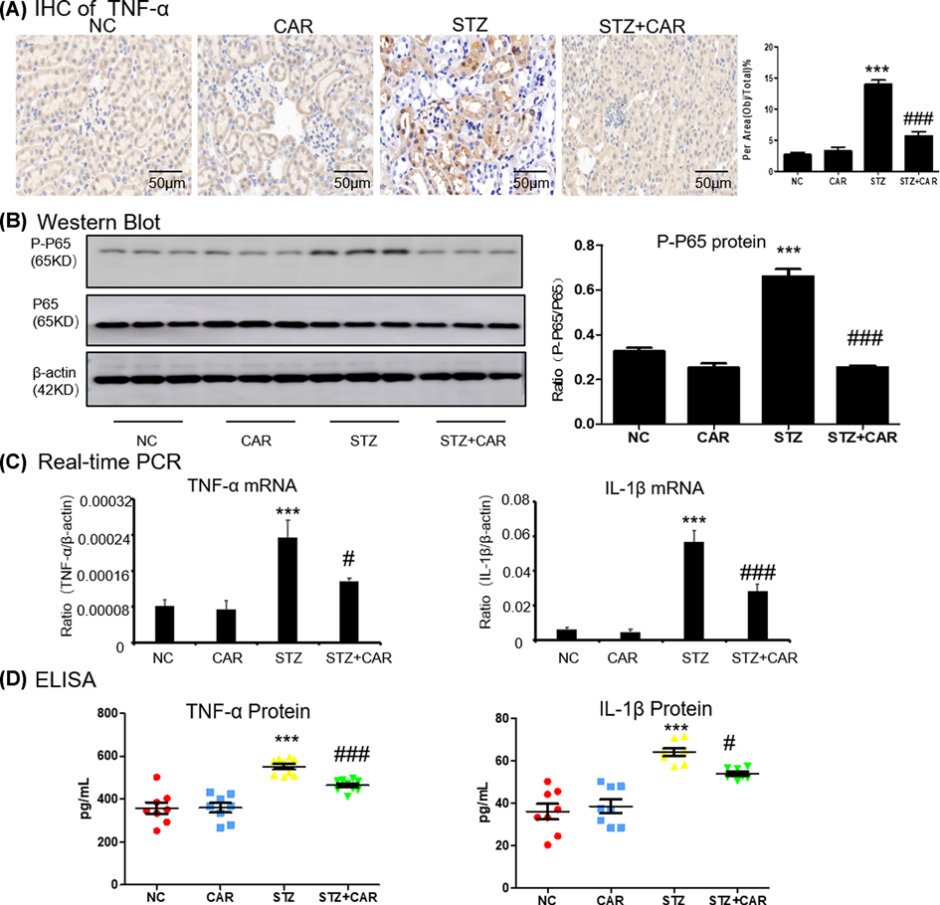
Carnosine attenuates renal inflammation in DN mice (**A**) Immunohistochemistry of TNF-a in mice kidney. (**B**) Western blot of P-P65 in mice kidney. (**C**) ELISA of inflammation indices in mice kidney. (**D**) Real-time PCR of inflammation indices in mice kidney. Data represent the mean ± SEM for 6–8 mice. Results represent means ± SEM for 6–8 mice. ***P<0.001 vs NC. #*P<*0.05, ###*P<*0.001 vs STZ; scale bar = 100 μm. Abbreviations: NC, normal control; CAR, carnosine.

**Figure 9:**
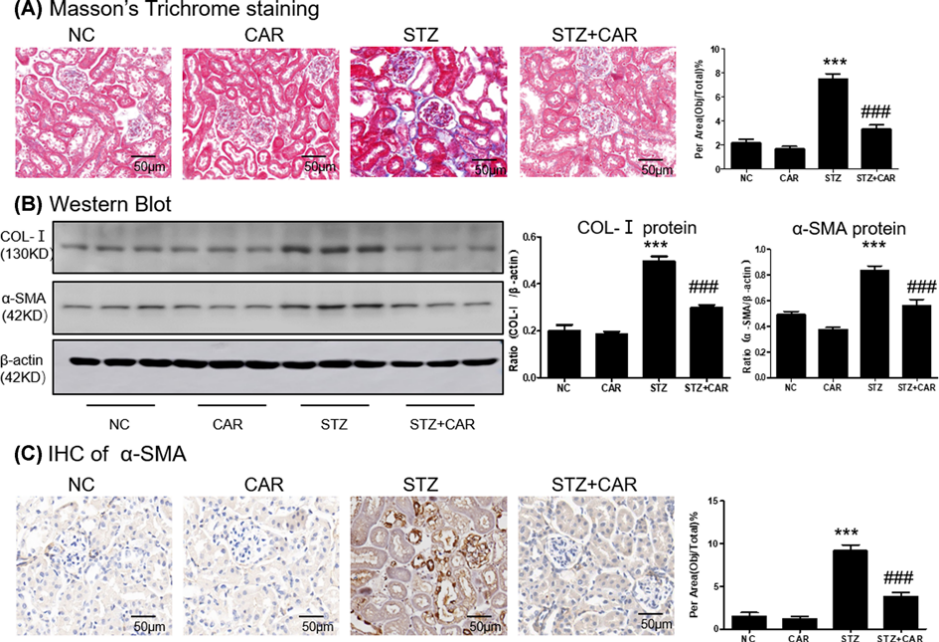
Carnosine attenuates renal fibrosis in DN mice (**A**) Masson's Trichrome staining and score of severity. (**B**) Western blot of Col-I and α-SMA in mice kidney. (**C**) Immunohistochemistry of α-SMA in mice kidney. Results represent means ± SEM for 6–8 mice. ***P<0.001 vs NC. ###*P<*0.001 vs STZ; scale bar = 100 μm. Abbreviations: NC, Normal control; CAR, carnosine.

We checked the anti-inflammatory effect of carnosine in DN mice and immunohistochemistry data showed that carnosine down-regulated the TNF-α level (Figure 8A). Results from western blot showed that carnosine suppressed phosphorylation level of P65 in DN mice (Figure 8B). Real-time PCR and ELISA showed that carnosine significantly reduced the levels of TNF-α, IL-1β (Figure 8C,D). Moreover, the accumulation of extracellular matrix (ECM) was the basic pathological feature of tubulointerstitial fibrosis. The histological observation of the fibrosis was confirmed by Masson trichrome staining, which showed a sharp increase in collagen deposition in the kidneys of DN mice at 16 weeks, while collagen deposition decreased in the carnosine-treated mice (Figure 9A and Supplementary Figure S1E). Moreover, carnosine also inhibited collagen I, and a-SMA (Figure 9B). This was consistent supported by results from immunohistochemistry of a-SMA (Figure 9C).

## Discussion

In the present study, the renoprotective role of carnosine associated with tubular epithelial cells inflammation and fibrosis was explored. Our findings indicated that carnosine could regulate the symptoms of DKD by relieving glomerular sclerosis, albuminuria and tubular epithelial cells damage *in vivo*. The possible mechanism was that carnosine could alleviate HG-induced tubular epithelial cells inflammation and ECM through targeting GNMT.

Carnosine is a bioactive peptide, which has the functions of buffering, regulating the body, scavenging free radicals, antioxidation, antiaging and preventing metabolic disorders [7,10–12]. Treatment with carnosine before ischemia can reduce the development of acute renal failure caused by ischemia/reperfusion, and inhibit the increase in renal norepinephrine release after ischemia/reperfusion [6,12]. Meanwhile, carnosine showed prevention of apoptosis of glomerular cells and podocyte loss in diabetic rats [8,12]. To better understand the renoprotective effects of carnosine, we detected clinical symptoms in 8-, 12- and 16-week diabetic mice, results show that albuminuria was significantly increased in an age-dependent manner and decreased after carnosine treatment; however, there was no significant reduction in blood glucose. Interestingly, we measured the levels of carnosine in the kidney and blood of mice, and the results showed that after carnosine treatment, renal and serum carnosine levels increased significantly, but not in an age-dependent manner. Furthermore, in diabetic mice, increased with age, kidney fibrosis gradually worsens, and carnosine shows the best antifibrotic effect at 16 weeks. Here, we showed that carnosine plays a key role in alleviating renal inflammation and fibrosis in DN by targeting GNMT. Our results also highlighted that the dysregulated GNMT is closely linked HG-induced MTEC injury.

In the present study, we showed the anti-inflammatory activity of carnosine on HG-induced cell damage. Further comprehensive investigation in the present study, we found that carnosine treated STZ-induced diabetic mice and significantly inhibited inflammation. Results showed that carnosine reduced the levels of TNF-α, IL-1β, and P65 phosphorylation. With the deepening of studies on DN, it is now generally believed that DN is a chronic inflammatory disease, in which the structure of glomerulus and renal tubules are changed by long-term micro-inflammation, which leads to proteinuria [13,14]. Studies have shown that factors such as high glycemic environment promote the activation of NF-κB signaling pathway and TNF-α, MCP-1, IL-6 and IL-1β, the release of IL-1β and other inflammatory factors contributed to the renal injury of DN [15]. Specific blocking of the above signaling pathways or inflammatory cytokines can reduce renal inflammation and cell injury in DN mice, thereby reducing DN [15,16].

Consistent with above, our data indicated that the carnosine treatment reduced renal fibrosis. It has been shown that carnosine could down-regulate COL-I, α-SMA and fibronectin. The main manifestations of renal fibrosis include exudation of inflammatory cells, activation and proliferation of fibroblasts, mass production of extracellular matrix, loss of renal intrinsic cells and reduction of microvessels [17,18]. It is important to note that the inflammatory response runs through the entire process of renal fibrosis. A large amount of pathological evidence showed that the fibrotic lesions were often distributed along the blood vessels [19,20]. Inflammatory microenvironment is closely related to endothelial function injury: the higher the concentration of local inflammatory factors, the more active fibrous hyperplasia, suggesting the important role of inflammatory response in renal fibrosis [21,22]. To our knowledge, this is the first study to demonstrate that carnosine could reduce fibrosis on STZ-induced diabetes mice.

Additionally, we found that carnosine alleviates HG-induced apoptosis. Apoptosis is a strictly controlled cell death process, which is necessary for the development of organisms and cell growth. Apoptosis pathways are involved in cell growth and differentiation in many diseases including DN [23]. In the inflammatory response, the cytoplasmic double-stranded DNA associated with the pathogen and the host can activate inflammatory factors, and then activate caspase to induce the maturation of the precursor of IL-1β and promote inflammation [24–26].

The results of the present study showed that carnosine has an effective protective effect on the kidney; we further determined its mechanism of action. We used DS software to predict the molecular target of carnosine. Through computer-aided simulation, the interaction between carnosine and GNMT was identified. Target engagement (TE) is an important factor in evaluating the development potential of drugs. The Cellular Thermal Shift Assay (CETSA) can measure intracellular TE at various stages of drug development. Carnosine binding to GNMT was confirmed further by CETSA. Further, molecular docking was used to clarify and analyze the most stable binding posture on GNMT active sites. Additionally, increased GNMT mRNA levels observed in response to carnosine in the HG-treated group may be the result of an indirect effect of decreased inflammation leading to higher GNMT level in a positive feedback loop and warrants further investigation.

The role of GNMT in chronic kidney disease such as DKD has never been reported previously. Interestingly, we first found that GNMT was down-regulated observably in the serum of DN patients. GNMT is an enzyme dependent on S-adenosine l-methionine (SAM) that catalyzes the conversion of glycine to creatine [27–29]. SAM-dependent methyltransferases are inhibited by S-adenosyl-L-homocysteine (SAH), and GNMT is believed to play a key role in other methyl transfer reactions as a regulator of the cell SAM/SAH ratio [30,31]. GNMT is enriched in liver, kidney and other organs and plays an important role in cell protection [32]. In liver diseases, AAV8-GNMT significantly reduces the level of profibrosis markers and improves the proliferation efficiency of hepatocytes [27]. Moreover, down-regulation of GNMT leads to loss of liver function and progression to fibrosis, cirrhosis and hepatocellular carcinoma, and lack of GNMT aggravates fibrosis caused by cholestasis [33]. Additionally, in diabetic kidneys, increased SAM levels in renal tubules associated with reduced GNMT expression and unrestricted methionine intake contribute to the activation of the target of rapamycin complex 1 (mTORC1) mechanism and impaired autophagy [34]. However, whether GNMT can regulate fibrosis and inflammatory response in diabetic nephropathy has not been reported. At the same time, it is urgent to explore whether GNMT can be used as a potential therapeutic target in diabetic nephropathy.

Our study demonstrates that down-regulation of GNMT protein level in STZ-induced diabetic mice kidney was observably reversed by carnosine intervention. Moreover, the present study identifies that GNMT overexpression attenuated HG-induced renal inflammation and fibrosis. However, results showed that carnosine had no further remission in HG-induced high levels of production of inflammatory factors and cell injury in GNMT knockdown cells. Importantly, the above results found that GNMT can be regarded as a new target for the treatment of inflammation and fibrosis in diabetic nephropathy. Current results suggest that carnosine may exert anti-inflammatory and antifibrotic effects in STZ-induced Type 1 diabetes experimental mouse models by targeting GNMT. Indeed, considering the low cytotoxicity of carnosine, it may be a promising new agent for the treatment of DN.

In conclusion, we demonstrate renoprotective effect of carnosine on STZ-induced diabetic mice by targeting GNMT-dependent inflammation and fibrosis. Therefore, further exploration of GNMT analogs and other drugs based on the interaction of carnosine and GNMT may help to find more effective and safer drugs, and then conduct clinical trials in DKD patients.

## Clinical perspectives

- Diabetic nephropathy (DN) is a common microvascular complication of diabetes and there is still no effective treatment for DN.
- Carnosine protects clinical symptoms and attenuates inflammation and fibrosis in DN by interacting with GNMT, a newly identified anti-inflammatory enzyme in DN.
- Carnosine is a novel GNMT agonist with high efficiency in treatment of DN and GNMT may serve as a potential therapeutic target for DN.

## Supplementary Material

Supplementary Figure S1

## Data Availability

The finding of carnosine binding directly to GNMT is supported by computational model (the Protein Model Database http://srv00.recas.ba.infn.it/PMDB/) and the PMDB ID is PM0083417.

## Abbreviations

BUN: blood urea nitrogen
CAR: carnosine
Cr: creatinine
CETSA: cellular thermal shift assay
DN: diabetic nephropathy
ECM: extracellular matrix
ESRD: end-stage nephropathy
GNMT: glycine N-methyltransferase
HG: high glucose
IF: immunofluorescence
KIM-1: kidney injury molecule-1
MTECs: mice tubular epithelial cells
PAS: Periodic acid–Schiff
SAM: S-adenosylmethionine
SAH: S-adenosylhomocysteine
STZ: streptozotocin
