# Supplementary material for: Carnosine alleviates diabetic nephropathy by targeting GNMT, a key enzyme mediating renal inflammation and fibrosis
Source: Clin Sci (Lond). 2020 Dec 9;134(23):3175–93. doi: 10.1042/CS20201207 (PMC7726623; doi:10.1042/CS20201207)
Supplement: Supplementary Figure S1 [file CS-2020-1207_supp.pdf]

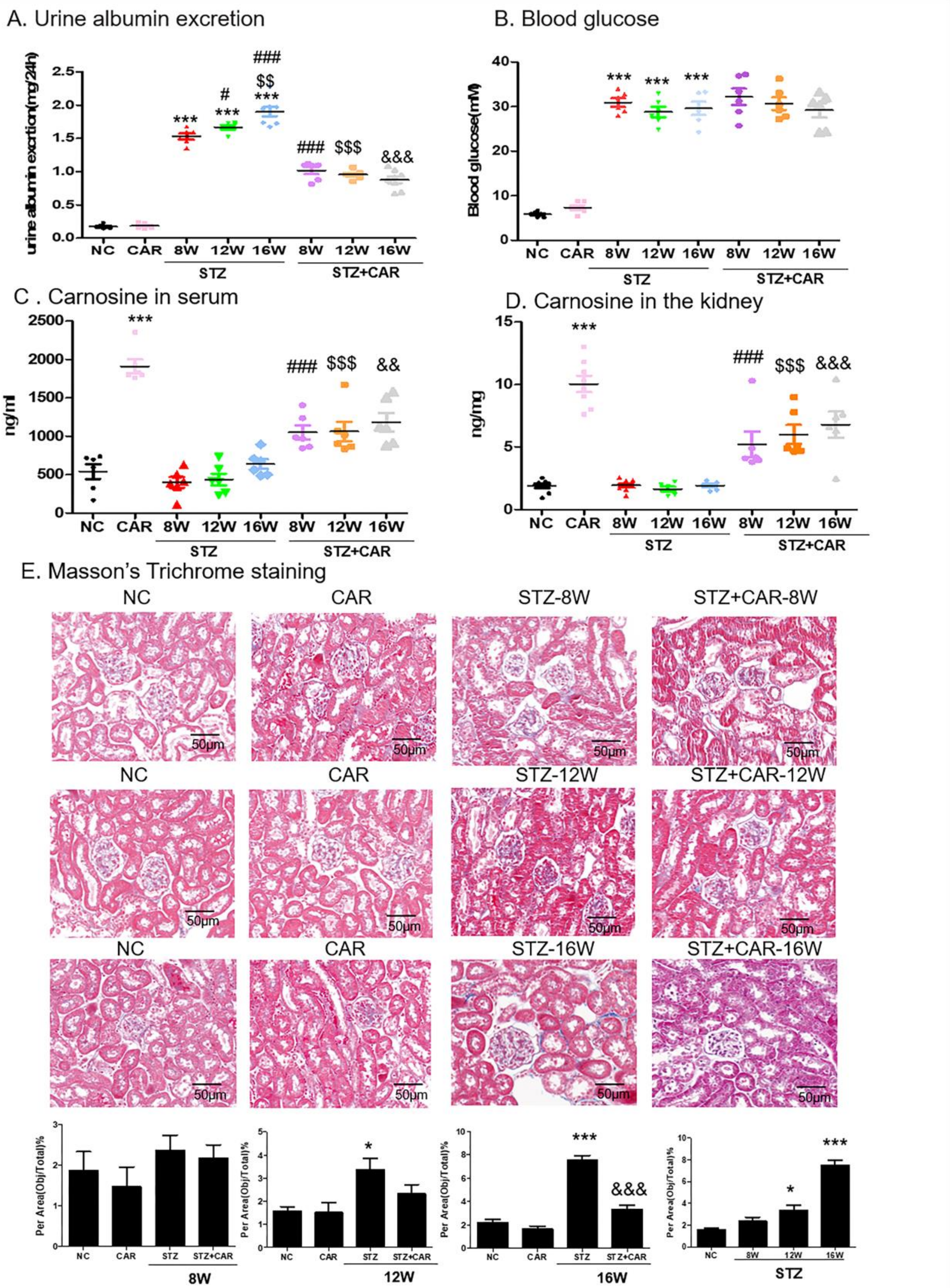

Supplement Figure1

Supplement Figure 1. Physical and biochemical markers and pathology in DN mice at 8, 12 and 16 weeks.

(A)Analysis of urine albumin excretion. (B)Blood glucose. (C) The concentration of carnosine in mice kidney. (D) The concentration of carnosine in serum. (E) Masson’s Trichrome staining and score of severity. Results represent means ± SEM for 6–8 mice. *p< 0.05, **p< 0.01, ***p< 0.001 VS NC. #p <0.05, ##p <0.01, ###p <0.001 VS STZ-8W. $p< 0.05, $$p< 0.01, $$$p< 0.001 VS STZ-12W.&p< 0.05, &&p< 0.01, &&&p< 0.001 VS STZ-16W. Scale bar = 100μm. Abbreviation: NC, Normalcontrol. CAR, carnosine.
